# Effect of Japanese Kampo medicine, eppikajutsuto, in patients with lymphatic malformation: A retrospective observational study

**DOI:** 10.1097/MD.0000000000028420

**Published:** 2021-12-23

**Authors:** Keiko Ogawa-Ochiai, Keigo Osuga, Taiki Nozaki, Yuko Tazuke, Seisho Sakai, Shuichiro Uehara, Reina Hoshi, Hideki Ishikawa, Kenichi Yoshimura, Hiroomi Okuyama

**Affiliations:** a Kampo Clinical Center, Department of General Medicine, Hiroshima University Hospital, Hiroshima, Japan; b Department of Radiology, Osaka Medical College, Takatsuki, Osaka, Japan; c Department of Radiology, St Luke's International Hospital, Chuo-ku, Tokyo, Japan; d Department of Pediatric Surgery, Osaka University Graduate School of Medicine, Suita, Osaka, Japan; e Department of Pediatric Surgery, Kanazawa University Hospital, Kanazawa city, Ishikawa, Japan; f Department of Pediatric Surgery, Nihon University School of Medicine, Itabashi-ku, Tokyo, Japan; g Department of Molecular-Targeting Cancer Prevention, Graduate School of Medical Science, Kyoto Prefectural University of Medicine, Chuo-ku, Osaka, Japan; h Department of Biostatistics, Medical Center for Translational and Clinical Research, Hiroshima University Hospital, Minami-ku, Hiroshima, Japan.

**Keywords:** eppikajutsuto, Kampo medicine, lymphatic malformation, vascular malformation, Yue-bi-jia-shu-tang

## Abstract

Lymphatic malformations (LMs) are congenital malformations of the lymphatic system that cause considerable cosmetic and functional complications. In this study, we present 8 children with LM who were treated with the Kampo medicine eppikajutsuto (EKJT).

Between 2001 and 2020, 8 children (male: 4, female: 4) with LMs who underwent magnetic resonance imaging (MRI) evaluation both before and after treatment or observation were selected for investigating the effect of EKJT. Two patients were observed without any treatment for 24 and 60 months. EKJT was evaluated based on percentage reduction, defined as the percentage of total lesions that decreased in size, confirmed by radiological examination after initiating treatment with EKJT or determined by observation alone. Volumetric analysis of LMs on MRI was performed using the Digital Imaging and Communications in Medicine viewer.

Six patients were treated with EKJT. The mean observational period was 13.2 months (range: 6–24 months). The mean reduction in LM volume on MRI was 73.0% in treated patients and –66.3% in observed patients. Two of the 6 lesions exhibited complete reduction, 2 exhibited marked (>90%) reduction, 1 exhibited moderate reduction, and 1 exhibited a small response. The treatment was well-tolerated, with no severe adverse events.

This preliminary study demonstrated the beneficial effects of EKJT. Prospective evaluations of this promising therapeutic modality are warranted based on the results of this study.

## 1 Introduction

Lymphatic malformations (LMs) are classified according to the International Society for the Study of Vascular Anomalies classification system (http://issva.org/classification). LM is defined as a congenital malformation of the lymphatic system that most often occurs in the head and neck region and can cause considerable complications, such as bronchial compression, particularly in children and neonates. It also occurs in the extremities, which can sometimes lead to a decrease in activities of daily living due to the limitation of motion or pain.

The incidence of LMs is unknown, but it is estimated to be 1 in 4000 livebirths (https://rarediseases.org/rare-diseases/lymphatic-malformations/#affected-populations, accessed on August 10, 2021). Sclerotherapy and surgical resection are the primary treatments for LM in the Japanese National Health Care System. Sclerotherapy is effective for cystic LM, but it can be difficult to perform because of the temporary swelling and compression of vital tissues around the lesion. Surgical resection is also challenging, and less invasive treatment is desired. Recently, sirolimus has been approved for general use as a treatment for LM in the United States and other countries,^[1]^ and several cases of LMs successfully treated with sirolimus have been reported in Japan.^[2]^ However, serious side effects have also been reported with sirolimus, including thrombocytopenia, edema, hyperglycemia, hyperlipidemia, lymphopenia, hypophosphatemia, infection, and cardiac problems. In addition, sirolimus is expensive, and prescriptions of 1308.8 yen/mg at 1 to 4 mg/d are common. Therefore, current standard therapies place a heavy burden on patients in terms of side effects and cost.

To the best of our knowledge, this is the first study to report the effect of eppikajutsuto (EKJT) on reducing lesion volume in mediastinal LM cases in Japan.^[3]^ We also reported a case with a marked reduction in cervical LM after increasing the dosage of EKJT.^[4]^ Other cases of LM reduction with EKJT have also been reported.^[5,6]^ However, no evidence has been established to evaluate its efficacy, dose dependency, and safety. LMs can cause life-threatening airway obstruction because they tend to swell and enlarge suddenly after a viral or bacterial infection and sclerotherapy with sclerosants such as OK-432. Since the amount of ephedrine contained in EKJT is very low, about 0.7% of the total amount of pseudoephedrine and ephedrine combined, it is considered safe to use under medical supervision and side effects of EKJT are very rare. EJT was approved by the Japanese Ministry of Health, Labour and Welfare in 1986, and is regulated by the Society of Japanese Oriental Medicine. (See http://mpdb.nibiohn.go.jp/stork). In the field of pediatrics, it has been used for many years for nocturia, and there have been no reports of serious side effects. The cost is remarkably low at 11 yen/g for 0.3 to 0.7 g/kg/d in children, going up to 7.5 g/d at 82.5 yen.

Kampo medicine is a traditional Japanese medicine. Its origin is in ancient China, but Kampo was developed under the influence of Japanese nature and culture. Kampo prescriptions are covered under the National Health Insurance Plan of Japan and are readily available to clinicians.

Recently, the application of Kampo to LMs has been reported, but there are no reports of its effects on the precise evaluation of radiological images. In this study, we prospectively investigated whether EKJT is effective for LMs by estimating the percentage reduction using MRI.

## 2 Materials and methods

### 2.1 Patient characteristics

Eight children with LMs (male: 4, female: 4; median age: 20.4 [6–60] months) were prospectively enrolled in this trial, which was approved by the Kanazawa University Institutional Review Board and conducted in accordance with the Declaration of Helsinki. The inclusion criteria were as follows: patients with all types of LMs, no history of sclerotherapy, surgery, or mTOR inhibitor administration, and MRI performed both before and after treatment. The exclusion criteria were as follows: patients who are receiving or have received mTOR inhibitors, patients who have undergone sclerotherapy or surgery during the observational term, patients for whom an evaluable MRI could not be obtained. We examined the charts of 386 patients, but only 8 patients fulfilled the inclusion and exclusion criteria.

### 2.2 Radiological evaluation

An experienced radiologist performed the imaging evaluations. The effect of EKJT was assessed based on percentage reduction, defined as the percentage of total lesions reduced in size by MRI volumetry at 6 to 24 months after initiation of EKJT treatment or evaluation by observation alone. The total volume of LMs demonstrated on fat-saturated T2-weighted MR images was measured using the Digital Imaging and Communications in Medicine viewer (OsiriX v.9.0; Pixmeo, Bernex, Switzerland). MR volumetry was semi-automatically performed to measure the area dimensions of the lesion slice by slice using the region of interest (ROI) tool. If ROIs could not be calculated because of the intricate shapes of the lesions, measurements were performed using a manual segmentation tool (closed polygon ROI). The volume of the target lesion was calculated by multiplying the ROI areas by the slice width.

### 2.3 Medication

EKJT (TJ-28, Tsumura & Co., Tokyo, Japan) at a dose of 0.2 to 0.3 g/d was administered orally to patients (n = 6) for a period of 6 months to 2 years. EKJT was approved by the Japanese Ministry of Health, Labour, and Welfare in 1986. It is also regulated by the National Institutes of Biomedical Innovation, Health and Nutrition (http://mpdb.nibiohn.go.jp/stork).

Two patients were followed without radical treatment during the observational term because there is no effect of LM on vital organs and the parents do not want active treatment. One of 2 cases was treated with sclerotherapy after the end of the study period. This case was treated with sclerotherapy after the study period.

### 2.4 Statistical analysis

Baseline values of MRI volumetry were measured on day 0 before administration. The difference in values at baseline and after EKJT administration was determined as the percentage reduction in MRI volumetry. The Wilcoxon signed-rank test was performed to compare the difference before and after the intervention. The data were considered to be statistically significant when the *P* value was <.05. Excel 2019 software (Microsoft, WA) was used for all statistical analyses.

## 3 Results

**Figure 1:**
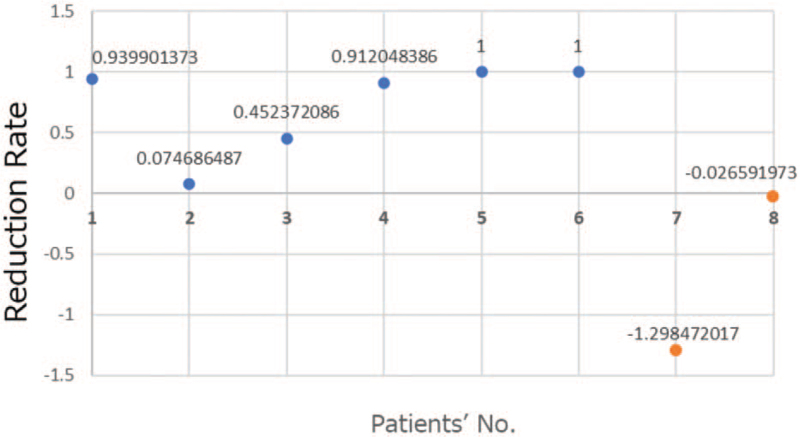
Volumetric changes shown using radiological examination in patients after eppikajutsuto treatment (blue) or observation alone (orange).

We reviewed 8 patients with LMs. Patient characteristics and treatment are summarized in Table 1. All patients had at least one target lesion that was measurable using MRI and were examined at 6 to 60 months. Volumetric changes are shown using radiological examination in patients in Fig. 1.

**Table 1:** Characteristics of the study patients.

N	Age at onset	Sex	Location of lesions	Dosing period, mo	Dose of EKJT, g/kg/d	Evaluation of radiological volumetric change (change rate, %)	Adverse effects associated with EKJT	Subsequent treatment
1	7 years	Male	Neck	6	0.25	94	None	Continuation of EKJT
2	5 years	Male	Right face and orbit	12	0.3	7.5	None	Surgery additional Kampo formula
3	4 months	Female	Neck and mediastinal	8	0.2	45.2	None	Continuation of EKJT
4	8 months	Female	Neck and mediastinal	15	0.3	91.2	None	Continuation of EKJT
5	10 months	Female	Neck	14	0.2	100	None	None
6	5 years	Female	Abdominal cavity	24	0.2	100	None	None
7	3 years	Male	Right upper extremity	24	None	−130	N/A	Surgery
8	6 years	Male	Light forearm	60	None	−2.7	N/A	Sclerotherapy

EKJT = eppikajutsuto.

**Figure 2:**
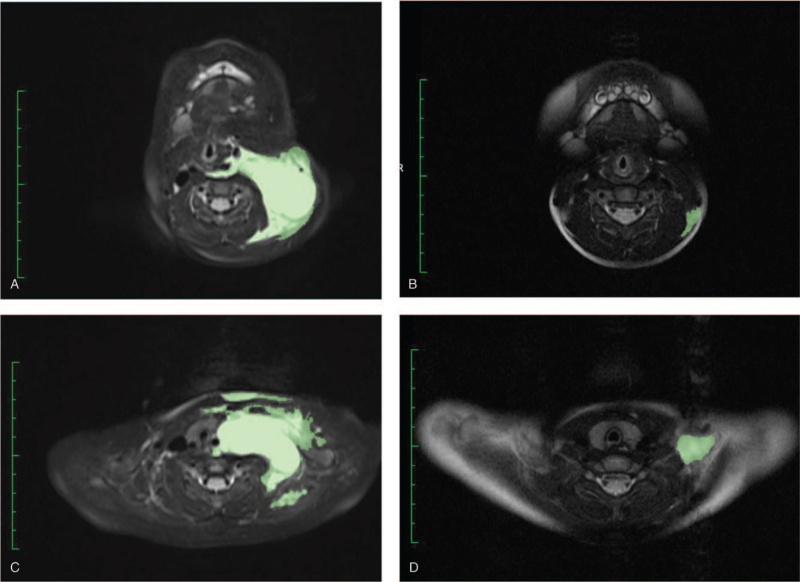
MRI of volumetric measurements for Case 4 (8-month-old woman with neck and mediastinal lymphatic malformation). T2-weighted MRI of the chest and neck demonstrates cystic lymphatic malformation, which is the target lesion. (A, C) Pretreatment; (B, D) 15 months after the administration of eppikajyutsuto. The volumetric measurements evaluated using the Digital Imaging and Communications in Medicine viewer (OsiriX) are also shown. MRI = magnetic resonance imaging.

Six patients were treated with EKJT. The mean observational period was 13.2 (range, 6–24) months. No patient discontinued treatment, and EKJT was generally well-tolerated without any severe adverse events. The MRI volumetry images of 2 of 6 patients are shown in Fig. 2.

Two cases were observed without any treatment for 24 and 60 months. The mean LM volume shrinkage on MRI was 73.0% in treated patients and –66.3% in observed patients. Two of the 6 lesions exhibited a complete reduction, 2 exhibited marked (>90%) reduction, 1 exhibited moderate reduction, and 1 exhibited a small response. The 2 observed patients had lesions in the upper extremities.

Among patients treated with EKJT, 5 had relatively large craniocervical lesions. One patient had abdominal lesions and experienced abdominal discomfort and pain. All 6 patients who underwent EKJT therapy exhibited a significant reduction in LM lesions (*P* = .03).

## 4 Discussion

We analyzed the effects of EKJT in the treatment of LM. Our study protocol used novel and precise radiological examination methods performed by experienced radiologists. All patients who received EKJT treatment displayed a reduction in the size of lesions, whereas those who did not receive EKJT manifested an increase in lesion size.

Kampo formulas are composed of multiple crude drugs, and each crude drug contains a wide variety of ingredients. EKJT is composed of 6 crude drugs. A 7.5 g portion of Tsumura EKJT extract granules contains 5.0 g of dried extract of the crude drugs listed in Table 2.

**Table 2:** Composition of eppikajyutsuto extract granules.

Constituents	Weight, g
Gypsum fibrosum	8
Ephedrae herba	6
*Atractylodis lanceae*	4
Zizyphi fructus	3
Glycyrrhizae radix	2
Zingiberis rhizoma	1

It is one of the most frequently used Kampo formulas in Japan, particularly for inflammatory disorders such as allergies and infections. The use of EKJT for the treatment of edematous lesions such as pterygium originated from an old medical book titled “Essentials from the Golden Cabinet,” which is the oldest clinical book dedicated to internal, external, gynecological, and obstetrical diseases. It was originally written by Zhang Zhongjing (Zhang Ji) (150-219 CE), an eminent Chinese physician in the Eastern Han dynasty. EKJT was suggested to reduce water congestion. Although it is difficult to fully understand the mechanisms of its effects, the results of our study indicate that EKJT downregulated the protein expression of mTOR but did not regulate ERK1/2 and phospho-ERK1/2 in OSC19 expression. We observed that cell proliferation inhibition and apoptosis induction depended on EKJT in vitro.^[7]^ It is possible that EKJT exerts an effect on LM through mTOR suppression. In a previous report, alkaloids of Ephedra Herba showed anti-inflammatory effects and CaSO_4_ of gypsum elevated aquaporin expression.^[8,9]^

Sclerotherapy is most commonly used to treat large and symptomatic macrocystic LMs in Japan because it is an easily repeatable and well-tolerated procedure without scar and low risk of direct nerve injury. Sclerosing agents, such as bleomycin, picibanil (OK432), doxycycline, sodium tetradecyl sulfate (STS), and alcohol, are injected percutaneously with ultrasound guidance.^[10,11]^ Sclerotherapy is most commonly done by outpatient interventional radiology, with the purpose of inducing fibrosis for reducing the mass of lesion. Risks include radiation exposure, skin necrosis, allergic reaction, and slow response to therapy. OK432 is the most common agent used for LM in Japan, induces inflammatory cytokines to reduce cystic lesions. Ohta et al^[10]^ reported 83 patients with cystic lesions of the neck injected with OK432 with resolution of 76% of lesions. Side effects included temporary pain, swelling, and low-grade fever in approximately half of patients.

Recently, sirolimus, a rapamycin (mTOR) inhibitor, offers promising treatment results in patients with complex LMs.^[12]^ Adverse effects are dose dependent and include hypertension, dyslipidemia, poor wound healing, bone marrow suppression (neutropenia, anemia, thrombocytopenia), and increased susceptibility to infections.

As described above, sclerotherapy and mTOR inhibitors have their own advantages and disadvantages, and the increase of treatment options for LM called EKJT improves patients’ ADL and QOL. In addition, EKJT administration is particularly suitable for patients in whom sclerotherapy is difficult due to the site of the LM or in whom there is concern about the side effects of mTOR inhibitors. Furthermore, EKJT is relatively inexpensive, leading to a reduction in medical costs.

This preliminary study demonstrated the beneficial effects of EKJT using novel and precise radiological examination methods. Although this was a retrospective study with different observational terms, the method of imaging evaluations which can be used for diffuse lesions is very precise to indicate that EKJT is one of the alternative for LM patients.

Prospective evaluations of this therapeutic modality with this method of imaging evaluations are warranted based on the results of this study. In Japan, EKJT has been used for lymphatic malformations since 2011, and there is not enough experience in its long-term use. It is necessary to study the effects and safety of long-term use in the future. Prospective studies to be conducted should include patients who have not yet received any other treatment immediately after LM diagnosis.

## Acknowledgments

The authors thank to Ms Ayako Kimura and Kumiko Matsuo for their assistance.

## Author contributions

Keigo Osuga, Yuko Tazuke, Shuichiro Uehara, Seisho Sakai, and Reina Hoshi selected the cases suitable for evaluation. Keigo Osuga and Kenichi Yoshimura designed the study. Taiki Nozaki and Keiko Ogawa-Ochiai performed the radiological diagnosis, and Taiki Nozaki analyzed the data. Keigo Osuga wrote the manuscript. Hiroomi Okuyama and Hideki Ishikawa supervised the findings of this work. All authors discussed the results and contributed to the final manuscript.

**Conceptualization:** Keiko Ogawa-Ochiai, Keigo Osuga, Seisho Sakai, Hideki Ishikawa, Kenichi Yoshimura, Hiroomi Okuyama.

**Data curation:** Keiko Ogawa-Ochiai, Keigo Osuga, Taiki Nozaki, Yuko Tazuke, Hideki Ishikawa.

**Formal analysis:** Taiki Nozaki, Kenichi Yoshimura.

**Funding acquisition:** Keiko Ogawa-Ochiai.

**Investigation:** Keiko Ogawa-Ochiai.

**Methodology:** Keiko Ogawa-Ochiai, Taiki Nozaki, Hideki Ishikawa.

**Project administration:** Keiko Ogawa-Ochiai, Yuko Tazuke, Seisho Sakai, Shuichiro Uehara, Reina Hoshi, Hideki Ishikawa.

**Resources:** Keiko Ogawa-Ochiai, Yuko Tazuke, Shuichiro Uehara.

**Supervision:** Keigo Osuga, Hideki Ishikawa, Hiroomi Okuyama.

**Writing – original draft:** Keiko Ogawa-Ochiai, Kenichi Yoshimura.

**Writing – review & editing:** Keiko Ogawa-Ochiai, Taiki Nozaki, Yuko Tazuke, Seisho Sakai, Shuichiro Uehara, Reina Hoshi, Hideki Ishikawa, Kenichi Yoshimura, Hiroomi Okuyama.
